# Discussion on the Mechanism of Gandoufumu Decoction Attenuates Liver Damage of Wilson's Disease by Inhibiting Autophagy through the PI3K/Akt/mTOR Pathway Based on Network Pharmacology and Experimental Verification

**DOI:** 10.1155/2023/3236911

**Published:** 2023-06-15

**Authors:** Lulu Tang, Chenling Zhao, Jing Zhang, Ting Dong, Huaizhen Chen, Taohua Wei, Jiuxiang Wang, Wenming Yang

**Affiliations:** ^1^Department of Neurology, The First Affiliated Hospital of Anhui University of Chinese Medicine, Hefei 230031, China; ^2^The First Clinical Medical College, Anhui University of Chinese Medicine, Hefei 230038, China

## Abstract

**Background:**

Gandoufumu decoction (GDFMD) is a traditional Chinese medicine that has been widely used to treat Wilson's disease (WD) liver damage patients. However, its specific molecular mechanism currently remains unclear. Autophagy as a key contributor to WD liver damage has been intensely researched in the recent years. Therefore, the aim of this present study is to explore the effect of GDFMD on autophagy in WD liver damage, and the final purpose is to provide scientific evidence for GDFMD treatment in WD liver damage.

**Methods:**

The molecular mechanisms and autophagy-related pathways of GDFMD in the treatment of WD liver damage were predicted using network pharmacology. Copper assay kit was used to determine copper content in serum. Enzyme-linked immunosorbent assay (ELISA) was utilized to quantify serum levels of liver enzymes and oxidative stress-related indicators. Hematoxylin-eosin (HE), Masson, and Sirius red staining were used for the characterization of liver pathological changes. Transmission electron microscopy, immunofluorescence, and Western blot analyses were used to evaluate autophagy activity. The impact of the GDFMD on typical autophagy-related pathway (PI3K/Akt/mTOR pathway) molecules was also assessed via Western blot analysis.

**Results:**

GDFMD effectively attenuated serum liver enzymes, oxidative stress, autophagy, and degree of hepatic histopathological impairment and reduced serum copper content. Through network pharmacological approaches, PI3K/Akt/mTOR pathway was identified as the typical autophagy-related pathway of GDFMD in the treatment of WD liver damage. Treatment with GDFMD activated the PI3K/Akt/mTOR pathway, an effect that was able to be counteracted by LY294002, a PI3K antagonist or Rapa (rapamycin), an autophagy inducer.

**Conclusions:**

GDFMD imparted therapeutic effects on WD through autophagy suppression by acting through the PI3K/Akt/mTOR pathway.

## 1. Introduction

Wilson's disease (WD) is an inherited metabolic disease culminating in pathological progressive copper accumulation in the liver and other tissues and is known to be caused by a genetic defect in the ATPase copper transporting beta gene (ATP7B) [1]. WD is a rare congenital disease, with prevalence rates ranging from approximately 1 : 10,000 to 1 : 30,000 worldwide. New studies have uncovered the existence of a significantly higher proportion of WD patients in China compared to the rest of the world [2]. Terminal WD is marked by liver cirrhosis and fibrosis, a clinically challenging entity to manage. It is to no surprise that this pathology and its related complications represent the primary cause of death in patients who suffer from WD [3]. Newer therapeutic options are vital in improving the survival of WD patients.

Autophagy is an omnipresent, highly conserved catabolic cellular recycling pathway and has been documented in organisms ranging from yeast to humans [4, 5]. Several types of stress conditions, such as energy deprivation, oxidative stress, protein aggregation, and tissue injury, have been shown to induce autophagy, an event that is central in cellular survival and homeostasis [4]. Dysfunctional autophagy has been linked to a myriad of diseases, such as cancer [6], liver-related diseases [4], metabolic disorders [7], neurodegenerative disease [8], and cardiovascular diseases [9]. Autophagy as a key contributor to WD liver damage has been intensely researched in the recent years [10]. Polishchuk et al. demonstrated an increase in autophagy activity in the livers of ATP7B^−/−^ rats as well as in those of WD patients. Moreover, in vitro experiments reveal that high cellular copper loads trigger autophagy [11]. Regulation of autophagy can delay the pathological progression of WD liver damage.

Traditional Chinese medicine (TCM) has been trialed as a novel WD therapy for several years. Investigations on TCM highlighted its abilities to reduce hepatic fibrosis, enhance urinary copper excretion, and protect the kidneys and brain [12]. A TCM product of interest in this study is the Gandoufumu decoction (GDFMD), which according to TCM theory can tonify the liver and kidney, nourish Yin energy, and resolve phlegm and blood stasis. The GDFMD comprises of *Reynoutria multiflora (Thunb.) Moldenke*, *Lycium barbarum L*., *Panax notoginseng (Burkill) F.H.Chen*, *Curcuma aromatica Salisb*., *Smilax glabra Roxb*., *Bupleurum chinense DC*, and *Paeonia lactiflora Pall*. Our group has demonstrated that the GDFMD could improve serological markers of liver function as well as the spleen pachydiameter value and liver ultrasound integral in patients with WD-mediated liver injury [13]. GDFMD has been commended to treat liver damage in the “Guidelines for Diagnosis and Treatment of Wilson's Disease with Integrated Traditional and Western Medicine” in China (2021). Nevertheless, the mechanism of GDFMD in WD liver damage requires further investigation.

In the past decade, with the development of bioinformatic methods, network pharmacology has become a dominant paradigm in investigating the multitarget pharmacological effects and complicated action mechanisms of TCM [14–16]. This approach provides direct visualization of the multitarget effects of TCM, consistent with the holistic and synergistic characteristics of the TCM theory [17, 18]. In the current study, the possible targets and signaling pathways were predicted using the network pharmacology method, which were then verified by in vivo and in vitro experiments to explore the potential hepatic protection mechanism of GDFMD more reasonably and provide a new direction for WD liver damage intervention and therapies.

## 2. Materials and Methods

### 2.1. Drug and Disease-Relevant Target Acquisition

The active compounds of each herb in the GDFMD formula were obtained by searching the Traditional Chinese Medicine Systems Pharmacology (TCMSP, https://tcmspw.com/tcmsp.php) database and related literature [19, 20]. We set the cutoff of oral bioavailability (OB) ≥ 30% and drug-likeness (DL) ≥ 0.18 in TCMSP as the threshold for the active compounds [21]. The UniProt (https://www.uniprot.org/) database and Perl language were used to query gene targets corresponding to the active compounds of GDFMD [22]. WD liver damage-related genes were mined from the GeneCards (https://www.genecards.org/) database. The intersection between the targets of active compounds in GDFMD and WD liver damage-related genes was visualized using VENNY 2.1 (https://bioinfogp.cnb.csic.es/tools/venny/) online platform.

### 2.2. PPI and TCM Compound Regulation Network Establishment

The intersection targets were used to establish a protein-protein interaction (PPI) network by the STRING database (https://string-db.org/) and Cytoscape software (version 3.8.2) [23, 24]. The species was limited to “Homo sapiens,” with the minimum required interaction score set to the “highest confidence of 0.7.” The disconnected nodes in the network were hidden to obtain a PPI network between intersection targets. The nodes represent targets, and edges represent the interactions between the targets. Simultaneously, the TCM compound regulation network was established to explain the molecular association between GDFMD and WD liver damage.

### 2.3. Enrichment Analysis

To clarify the possible biological functions and key pathways of GDFMD in the treatment of WD liver damage, the R software (version 4.2.1) was used to perform ID conversion on the names of the intersection targets, following which Gene Ontology (GO) and Kyoto Encyclopedia of Genes and Genomes (KEGG) enrichment analyses were carried out. Moreover, the top ten items with the smallest *P* values for the biological processes (BP), cellular components (CC), and molecular functions (MF) were selected to plot circle graphs, and the top thirty items with the smallest *P* values for the KEGG signaling pathways were selected to plot bubble charts. As *P* represents the significance of enrichment, the screening condition was limited to *P* < 0.05.

### 2.4. Preparation of GDFMD

The GDFMD comprises of 4 g of *Reynoutria multiflora (Thunb.) Moldenke*, 20 g of *Lycium barbarum L*., 3 g of *Panax notoginseng (Burkill) F.H.Chen*, 12 g of *Curcuma aromatica Salisb*., 12 g of *Smilax glabra Roxb*., 12 g of *Bupleurum chinense DC*, and 15 g of *Paeonia lactiflora Pall*. All herbs were supplied by the pharmacy of the First Hospital Affiliated to Anhui University of Chinese Medicine (Hefei, China). The medicinal herb mixture was immersed in water (10 times the weight of medicine) for 1 h, boiled twice for 1 h, and then concentrated to 0.2/mL to obtain GDFMD suspension [25].

### 2.5. Animals and Grouping

C3HeB/FeJ^Atp7btx−J^/J and control C3HeB/FeJ mice were procured from the Experimental Animal Center of the Jackson Laboratory (Bar Harbor, ME, USA). All mice weighed between 20 and 40 g and were reared in specific-pathogen free (SPF) conditions in temperature-controlled standardized cages under a 12 h : 12 h light : dark cycle at the Academy of Life Sciences of Anhui Agricultural University (Anhui, China). Animals were grouped into 5 cohorts (*n* = 10 per group) as follows: model, Gandoufumu (high dose, middle dose, and low dose), and penicillamine (PCA) cohorts (Shanghai Shangyaoxinyi Pharmaceutical Co., Ltd., China).

Intragastric administrations of 0.2 mL/10 g normal saline were given to the control and model groups. Mice in the PCA and Gandoufumu groups were treated with daily doses of 0.1 g/kg PCA and GDFMD (13.92, 6.96, and 3.48 g/kg), respectively, for a total of 28 successive days. At the end of the treatment period, all mice were anesthetized with sodium pentobarbital (50 mg/kg, i.p.), and venous blood was collected from the retroorbital sinus, and liver tissue was excised and rinsed with 0.9% NaCl solution. The Animal Ethics Committee of Anhui University of Chinese Medicine approved all protocols regarding animal care and experiments that were used in this study (2018AH-08).

### 2.6. Histological Analysis

Liver samples were treated with 4% formaldehyde prior to production of 5 *μ*m sections for staining with hematoxylin-eosin (HE) and Masson's trichrome. Structural liver changes were then visualized under an optical microscope (Eclipse E100, Nikon, Tokyo, Japan). The liver damage score was assessed by three separate pathologists based on the following: 0: no damage; 1-3: mild damage; 4-6: intermediate damage; 7-9: severe damage; and 10: damage of liver architecture [26]. Sirius red stains were used to assess the extent of histological liver fibrosis. Staining liver samples were scanned with the ImageJ software.

### 2.7. Determination of Serum Copper Content

Serum samples were prepared, and serum copper content was determined according to the copper assay kit instructions (complexation colorimetry). The kit was purchased from the Jiancheng Biotechnology Research Institute (Nanjing, China).

### 2.8. Cell Culture and Treatment

HepG2 cells were purchased from the Wuhan Pu-nuo-sai Life Technology Co. Ltd. (Wuhan, China), maintained in with foetal bovine serum (Tianjin Kang Yuan Biological Technology Co., Ltd., China) (20%), and supplemented minimal essential medium (Wuhan Pu-nuo-sai Life Technology Co. Ltd., China), which also contained penicillin/streptomycin (1%). Cells were cultured at 5% CO_2_ in a humidified tank at 37°C.

Treated mouse serum samples were prepared by extracting blood from the abdominal aorta, which was then centrifuged for 20 minutes at 3500g. The supernatant was incubated for 30 min in a 56°C water bath before being filtered with a 0.22 *μ*m microporous membrane (Millipore, USA). The resultant GDFMD-containing serum was maintained at −80°C until further use. Serum of 10% GDFMD concentration was used for further experiments [27].

For studying the modulatory effect of GDFMD in HepG2 cells, copper chloride (CuCl_2_) in 100 *μ*M was used as the model group [27]. HepG2 cells were then divided to form 6 groups: control, model, GDFMD, 3-MA, GDFMD+LY294002, and GDFMD+rapamycin (Rapa). 3-MA, LY294002, and Rapa were supplied by Sigma-Aldrich (St. Louis, MO, USA). Cells were allowed to incubate for 24 hours with their allocated treatment and were subsequently subjected to transmission electron microscope, immunofluorescence staining, Western blot, and ELISA.

### 2.9. Transmission Electron Microscope

Cells were fixed in 2.5% glutaraldehyde (Macklin Biochemical Co., Ltd., China) and sectioned (thickness, 50-100 nm) for observation under a transmission electron microscope (H7100, Hitachi Co., Ltd., Tokyo, Japan).

### 2.10. Immunofluorescence Staining

Cells were stained for immunofluorescence on coverslips. Cells were first fixed and permeablized before they were exposed to primary antibodies against LC3A/LC3B (1 : 100) overnight at 4°C and secondary antibodies at 37°C for 50 minutes. Slides were rinsed with PBS thrice and counterstained with DAPI. A positive fluorescence microscope (Nikon Eclipse C1, Tokyo, Japan) was used to visualize DAPI fluorescent signals.

### 2.11. Western Blot

RIPA lysis buffer was used for total protein extraction from liver tissues and HepG2 cells. Protein concentrations were measured with a BCA protein assay kit. 10 *μ*g total protein was first electrophoresed with a 5% SDS-PAGE prior to Western blotting that was performed based on standard protocols. *β*-Actin protein was used as a reference. Primary antibodies were used as follows (all diluted at a ratio of 1 : 1000 unless otherwise specified): Anti-PI3K, anti-phospho-PI3K, anti-Akt, anti-phospho-Akt (1 : 2000 dilution), anti-mTOR, anti-phospho-mTOR, anti-LC3A/LC3B, anti-p62, anti-beclin-1, *β*-actin, goat anti-rabbit, and goat anti-mouse-IgG HRP. All antibodies were purchased from Cell Signaling Technology Inc. (Danvers, MA, USA). The ImageJ software was used to analyze band densities (US National Institutes of Health, Bethesda, MD).

### 2.12. ELISA

The levels of ALT, AST, ALB, TP, ROS, SOD, MDA, and GSH-Px were quantified by ELISA based on protocols stipulated by the manufacturer.

### 2.13. Statistical Analysis

All quantitative data is depicted in terms of mean ± standard deviation (SD) and processed using SPSS version 22.0 (SPSS Inc., Chicago, IL, USA). Significant differences were analyzed using the one-way analysis of variance (ANOVA), and *P* values less than 0.05 were interpreted to be statistically significant.

## 3. Results

### 3.1. Analysis of Drug and Disease-Relevant Targets

A total of 67 active compounds of GDFMD were screened from the TCMSP database and related literature corresponding to the 254 potential targets. After converting the drug targets to standard gene names using UniProt database and Perl language, a total of 215 gene targets of GDFMD were obtained. In addition, a total of 687 WD liver damage-related targets were retrieved from the GeneCards database.

### 3.2. Construction Results of PPI and TCM Compound Regulation Network

**Figure 1:**
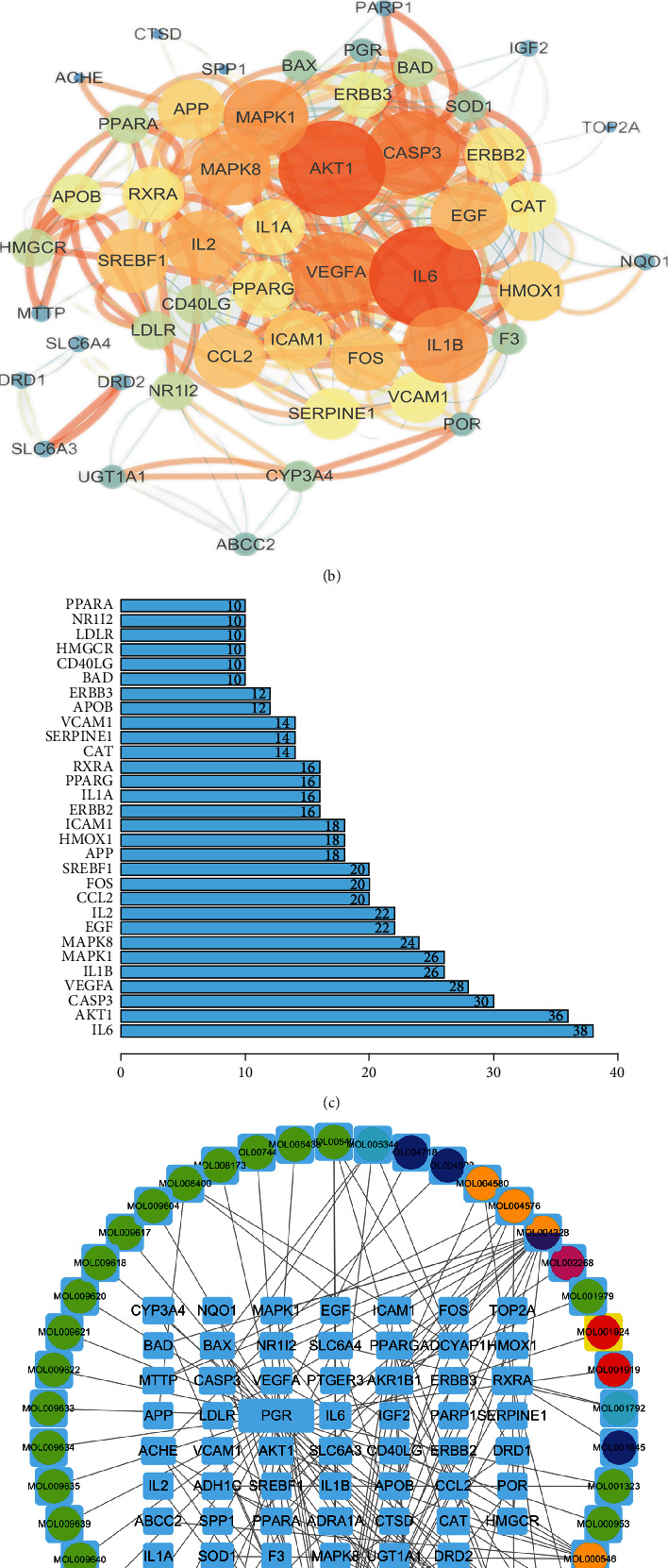
Network pharmacology analysis of GDFMD in the treatment of WD liver damage. Venn diagram (a); purple circles represent the gene targets of GDFMD, and yellow circles represent the gene targets of WD liver damage. PPI network based on STRING database (b); the more lines, the larger circle, and the brighter color represent the stronger correlation. Core genes in PPI network (c). TCM compound regulation network (d); light blue indicates intersection targets, leaf green indicates *Lycium barbarum L.*, cyan indicates *Panax notoginseng (Burkill) F.H.Chen*, baby blue indicates *Bupleurum chinense DC*, yellow indicates *Smilax glabra Roxb.*, purple indicates *Curcuma aromatica Salisb.*, rose indicates *Reynoutria multiflora (Thunb.)*, and red indicates *Paeonia lactiflora Pall*.

Using VENNY 2.1 online platform, a total of 55 intersection targets between GDFMD and WD liver damage were presented (Figure 1(a)). PPI network among these intersection targets was constructed based on STRING database. After topological analysis, the intersection targets were labeled with corresponding colors according to their node degrees (Figure 1(b)) and further analyzed using the R software (version 4.2.1) to identify the top 30 core genes of GDFMD in the treatment of WD liver damage (Figure 1(c)). Subsequently, the TCM compound regulation network was constructed for illustrating the complex interactive relationship between compounds and targets, and the edges represent the holistic and synergistic interaction between GDFMD and WD liver damage (Figure 1(d)).

### 3.3. Prediction Results of GO and KEGG Enrichment Analyses

**Figure 2:**
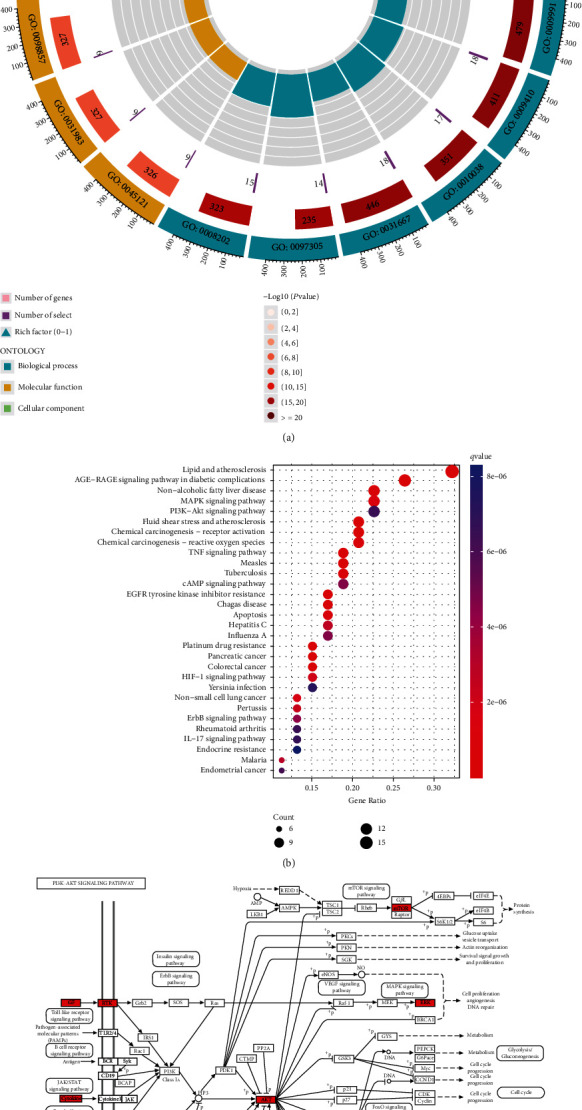
Enrichment analysis of GDFMD in the treatment of WD liver damage. Top six BP, CC, and MF items extracted from GO enrichment analysis (a). Top 30 pathways extracted from KEGG enrichment analysis (b). Representative enriched KEGG pathway with key targets labeled red (c).

A total of 1935 GO biological function items were obtained. The BP mainly involved response to extracellular stimulus, response to xenobiotic stimulus, and response to metal ion; CC mainly involved membrane raft, vesicle lumen, and membrane microdomain; MF mainly involved nuclear receptor activity, ligand-activated transcription factor activity, and signaling receptor activator activity (Figure 2(a)). A series of highly enriched KEGG pathways, such as lipid and atherosclerosis, AGE-RAGE signaling pathway in diabetic complications, nonalcoholic fatty liver disease, MAPK signaling pathway, and PI3K-Akt signaling pathway (Figure 2(b)), was assigned to be the most promising pathways associated with GDFMD treatment of WD liver damage. The PI3K-Akt pathway has long been known to function as a classical autophagy-related signaling pathway, which key targets were labeled red (Figure 2(c)). Therefore, we verified the roles of PI3K/Akt/mTOR pathway in WD liver damage and the effect of GDFMD on PI3K/Akt/mTOR pathway in subsequent experiments.

### 3.4. Effect of GDFMD on Liver Damage and Serum Copper Content in WD Mouse Models

**Figure 3:**
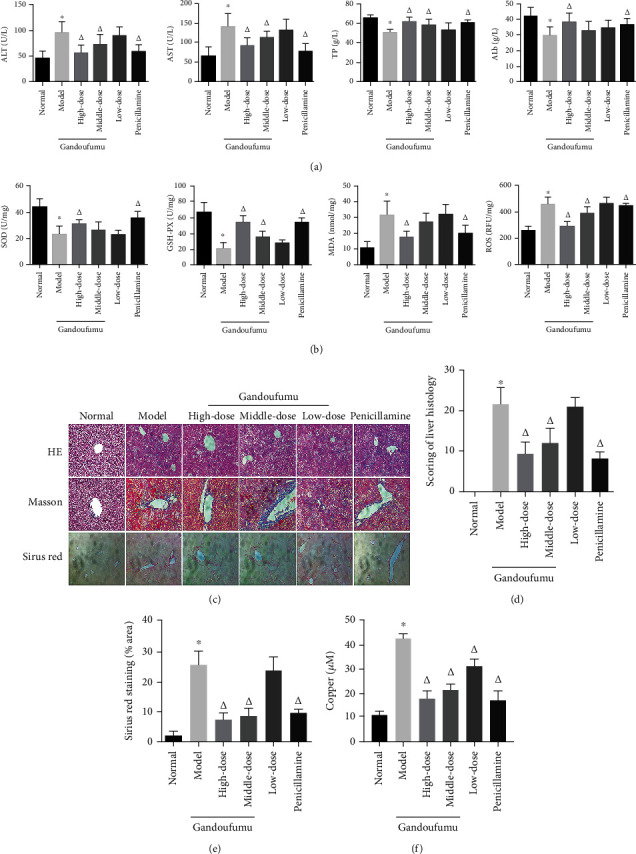
Effect of GDFMD on serum liver function, liver oxidative stress, pathological liver changes, and serum copper content of mouse models of WD. Serum levels of ALT, AST, TP, and ALB (a); liver oxidative stress of SOD, GSH-Px, MDA, and ROS was measured by ELISA assays (b). Representative photomicrographs of morphological changes (c) in HE (400x), Masson (400x), and Sirius red (400x) staining in liver tissues, scoring of liver histology (d), and quantification of Sirius red staining area (e) of mice from the control, model, Gandoufumu (high dose, low dose, and middle dose), and penicillamine groups. Serum copper content was determined by copper assay kit (f). Data is depicted in terms of mean ± SD (*n* = 10). ^∗^*P* < 0.05 compared with the normal group; ^△^*P* < 0.05 compared with the model group.

To explore the impact of GDFMD on the liver function in mouse models of WD, we examined the serum levels of ALT, AST, TP, and ALB. The ALT and AST levels were lowered, while the levels of TP and ALB were raised in the GDFMD and PCA groups (Figure 3(a)). Next, liver oxidative stress was assessed by quantifying MDA levels, ROS levels, and SOD and GSH-Px activities. As shown in the diagram (Figure 3(b)), the MDA and ROS levels were significantly lowered, while the levels of SOD and GSH-Px activities were raised in the GDFMD and PCA groups. Histopathological changes of the livers of mouse models of WD which were treated with GDFMD were observed with HE, Masson, and Sirius red staining to evaluate liver morphology. As illustrated in Figure 3(c), there was normal hepatic architecture with no fatty changes or inflammatory infiltration seen in the liver tissue of the control group. However, hepatic sections in the model group exhibited observable morphological aberrations of hepatocytes, notably fatty degeneration, necrosis, and vacuole formation. However, intragastric administration of GDFMD and PCA both significantly attenuated the severity of liver histological injury compared with those seen in the model group. Mice who were treated with GDFMD and PCA also demonstrated lower degrees of liver fibrosis, as evidenced by liver fibrosis scores and Sirius red staining patterns (Figures 3(d) and 3(e)). The copper assay kit was used to investigate the effect of GDFMD on serum copper content of WD mouse models. The copper content was significantly lowered in the GDFMD and PCA groups than that of the model group (Figure 3(f)). Collectively, these results indicate that GDFMD improved copper metabolism and had a protective effect against liver damage in WD mouse models.

### 3.5. Effect of GDFMD on Autophagy and Regulated Related Proteins of PI3K/Akt/mTOR Pathway in Mouse Liver Tissues

**Figure 4:**
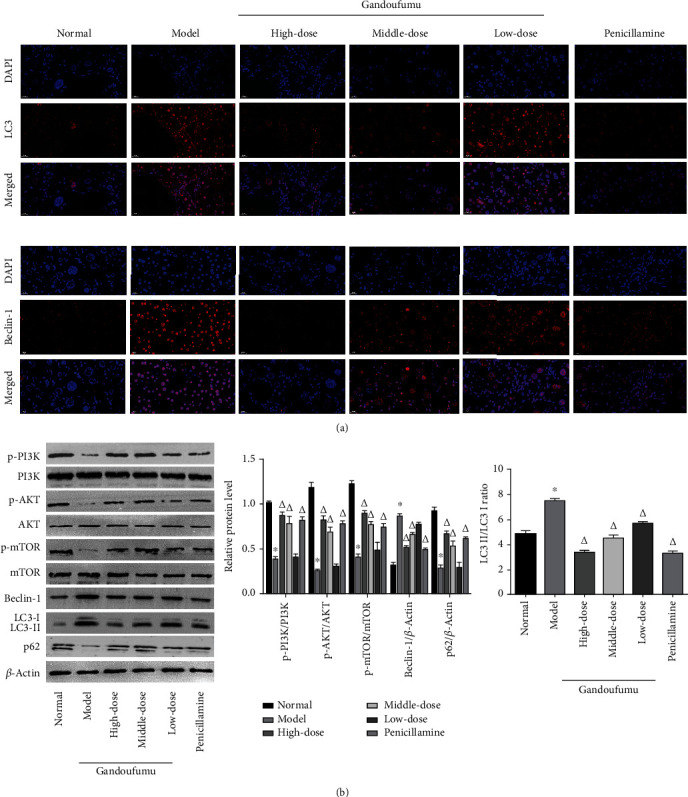
The effects of GDFMD on autophagy and PI3K/Akt/mTOR pathway-related proteins in the liver of mouse models of WD. Expression of LC3 and Beclin-1 was observed using immunofluorescence staining (a); the protein levels of p-PI3K, PI3K, p-AKT, AKT, p-mTOR, mTOR, Beclin-1, p62, and LC3-II/LC3-I ratio (b) in liver tissues of mice from the normal, model, Gandoufumu (high dose, low dose, and middle dose), and penicillamine groups. Data is depicted in terms of mean ± SD (*n* = 10). ^∗^*P* < 0.05 compared with the normal group; ^△^*P* < 0.05 compared with the model group.

Next, we evaluated the impact of GDFMD on autophagy in mouse models of WD. Based on immunofluorescence staining, there appeared to be a notable increase in immunostaining intensity of LC3 and Beclin-1 protein in liver tissues in the model group in contrast to controls; however, GDFMD and PCA treatments appeared to attenuate these changes (Figure 4(a)). Similarly, Western blot analysis of levels of autophagy-related proteins LC3-I, LC3-II, and Beclin-1 in liver tissues revealed them to be raised in the model group in comparison to those of the normal group. GDFMD and PCA-treated animals appeared to have lower levels of these proteins (Figure 4(b)). GDFMD appears to possess the ability to suppress autophagy in mouse models of WD. Interestingly, mice from the model group had suppressed levels of p-PI3K, p-Akt, and p-mTOR proteins, a finding that was reversed in groups treated with GDFMD and PCA. We conclude that PI3K/Akt/mTOR pathway activity modulation was a consequence of GDFMD administration in mouse models of WD.

### 3.6. Effect of GDFMD on HepG2 Cell Damage Induced by CuCl_2_

**Figure 5:**
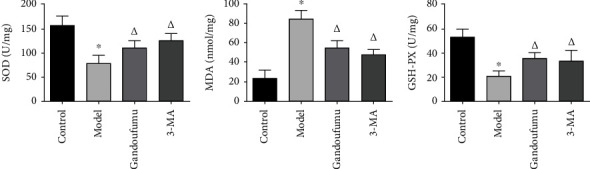
Effects of GDFMD on SOD, GSH-Px, and MDA levels in HepG2 cells were assessed using ELISA. Data is depicted in terms of mean ± SD (*n* = 3). ^∗^*P* < 0.05 compared with the normal group; ^△^*P* < 0.05 compared with the model group.

Further *in vitro* experiments were conducted to determine the effect of GDFMD on oxidative stress. With reference to Figure 5, CuCl_2_ stimulation increased levels of MDA while decreasing GSH-Px and SOD activities in HepG2 cells. GDFMD exposure appeared to mitigate this effect.

### 3.7. Effect of GDFMD on HepG2 Cell Autophagy and Regulated Related Proteins of PI3K/Akt/mTOR Induced by CuCl_2_

**Figure 6:**
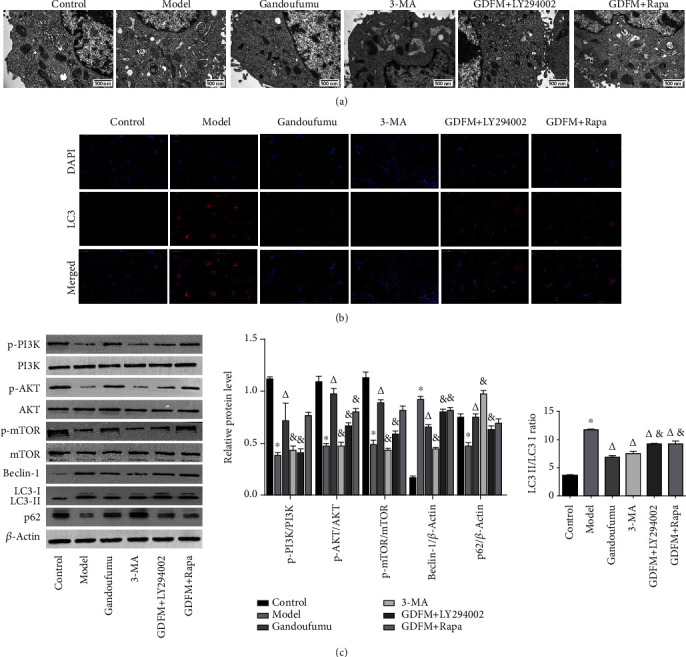
Effect of GDFMD on autophagy and PI3K/Akt/mTOR pathway-related proteins in HepG2 cells. Autophagosomes observed by transmission electron microscopy (a). LC3 expression visualized by immunofluorescence staining (b). The protein levels and determinations of p-PI3K, PI3K, p-AKT, AKT, p-mTOR, mTOR, beclin-1, p62, and LC3-II/LC3-I ratio (c) in the groups of control, model, Gandoufumu decoction, 3-MA, Gandoufumu+LY294002, and Gandoufumu+Rapa. Data is depicted in terms of mean ± SD (*n* = 3). ^∗^*P* < 0.05 compared with the normal group; ^△^*P* < 0.05 compared with the model group; ^&^*P* < 0.05 compared with the Gandoufumu group.

Autophagosomes were visualized in the HepG2 cells from each group using a transmission electron microscope. The number of autophagosomes was obviously increased in the model groups, while decreased after treatment with GDFMD or 3-MA, whereas increased in the LY294002 or Rapa group (Figure 6(a)).

Next, we observed LC3 protein expression in HepG2 cells using immunofluorescence analysis. LC3 proteins appear as an intracytoplasmic collection of red spots and was greatly raised in the model group in contrast to the control group. LC3 protein production appeared to be suppressed upon exposure to GDFMD and 3-MA, and further pretreatment with LY294002 or Rapa attenuated the inhibitory effect of GDFMD (Figure 6(b)).

In HepG2 cells, there was an upregulation of the LC3-II/LC3-I ratio and Beclin-1 protein levels, while p62 protein was a downregulated in the model group. These changes were reversed upon exposure to GDFMD. Blocking the PI3K pathway again had an inhibitory effect on the antiautophagic impact of GDFMD. CuCl_2_ exposure triggered suppression of PI3K, Akt, and mTOR phosphorylated protein levels. These effects were reversed with GDFMD which was in turn reversed again through the addition of LY294002 or Rapa (Figure 6(c)). These findings strongly support our hypothesis that GDFMD was involved in the PI3K/Akt/mTOR pathway modification in exerting its antiautophagic effects.

## 4. Discussion

WD is a genetic disease characterized by aberrant copper metabolism that presents with psychiatric, neurological, and hepatic derangements. Although clinical manifestations of WD vary widely, the most common ones are those of liver disease. Reports demonstrate that patients with WD often present with hepatic copper accumulation coupled with cirrhosis [28]. The recent years have proven TCM to be a viable therapeutic option in managing this condition. For example, Chen et al. demonstrated in mouse models of WD that Gandouling reduces the severity of cerebrovascular injury by working on the PERK/eIF2*α*/CHOP endoplasmic reticulum stress pathway [29]. Similarly, Gao et al. reported that the Gandou decoction was able to regulate the Wnt/*β*-catenin pathway, thereby reducing oxidative stress and alleviating the degree of liver injury in copper-loaded rats and copper-stimulated BRL-3A cells [30]. The GDFMD comprises *Reynoutria multiflora (Thunb.)*, *Lycium barbarum L*., *Panax notoginseng (Burkill) F.H.Chen*, *Curcuma aromatica Salisb*., *Smilax glabra Roxb*., *Bupleurum chinense DC*, and *Paeonia lactiflora Pall*., which has been reported to be effective in treating WD-related liver disease [13, 31, 32]. However, the underlying biological processes that mediate this therapeutic effect remain to be identified.

In the present study, network pharmacology-based approaches were used to identify the active compounds in GDFMD and the interactions between the GDFMD and WD liver damage-related genes. The further analysis (including PPI network, TCM compound regulation network, GO and KEGG enrichment) demonstrated that GDFMD plays an active role in the treatment of WD liver damage. The results of subsequent experiments show that the hepatoprotective effect of GDFMD in WD liver damage is brought about by inhibiting autophagy, which manifests as a lesser degree of histopathological liver damage, improved liver enzymes, reduced oxidative stress, and decreased serum copper content. In addition, our results uncovered that CuCl_2_-induced oxidative stress in HepG2 cells appeared to be alleviated by GDFMD. This compound also reduced autophagy through modulation of the PI3K/Akt/mTOR pathway activity.

Autophagy is a lysosomal self-digestion phenomenon that is vital in development, differentiation, and tissue remodeling across various organisms. A plethora of liver diseases has been shown to demonstrate deranged autophagy processes [33]. Autophagy acts as a double-edged sword where on one hand protecting hepatocytes from injury through elimination of damaged proteins and organelles but on the other acting as a pathway for accelerating liver pathology. For example, Ni et al. demonstrated that rapamycin-triggered autophagy was protective against the livers damaged by APAP; however, autophagy blockade by chloroquine in hepatocytes enhanced liver damage [34]. In contrast, Sharifi et al. demonstrated that tumor cells recruit energy through activation of autophagy, improving their survival ability under hypoxic and low-nutrient environments thereby promoting cancer progression in established hepatocellular carcinoma [35]. Our investigations revealed a strong association between WD-related liver injury and overwhelming autophagy levels, results that are in line with previously published studies. Additionally, GDFMD administration suppressed autophagy in the liver of WD mouse models, as evidenced by reduced formation of autophagosomes and improved expression of autophagy-related proteins, such as p62, Beclin-1, and LC3. Copper serves as a novel autophagy inducer, and its altered metabolism contributes to the pathogenesis of WD [36, 37]. Our results showed that treatment with GDFMD reduced copper accumulation in the serum, indicating that GDFMD has certain copper excretory effects.

The PI3K/Akt/mTOR pathway is notable for regulating autophagy in mammalian cells [38]. It appears that the PI3K/Akt/mTOR pathway negatively regulates autophagy as seen in WD [11, 39]. This effect has been documented to be significantly inhibited by TCM in *vitro* experiments [40, 41]. Similarly, this study revealed that GDFMD markedly reduced LC3 and Beclin-1 expressions while raising p62, p-PI3K, p-Akt, and p-mTOR expressions. We further investigated the antiautophagy molecular mechanism of GDFMD *in vitro*. CuCl_2_ promoted oxidative stress and autophagy of HepG2 cells by blocking the PI3K/AKT/mTOR pathway, while treatment of GDFMD reversed this effect. The antiautophagy effect of GDFMD was antagonized by LY294002 or Rapa.

In conclusion, the current investigation demonstrated that GDFMD significantly protects against WD liver damage by inhibiting overwhelming cellular autophagy through activation of the PI3K/Akt/mTOR signaling pathway.

## Data Availability

Data is available at Traditional Chinese Medicine Systems Pharmacology (TCMSP, http://lsp.nwu.edu.cn/tcmsp.php) database, UniProt (https://www.uniprot.org/) database, GeneCards (https://www.genecards.org/) database, and VENNY 2.1 (https://bioinfogp.cnb.csic.es/tools/venny/) online platform.
