# Correction to “Discussion on the Mechanism of Gandoufumu Decoction Attenuates Liver Damage of Wilson’s Disease by Inhibiting Autophagy through the PI3K/Akt/mTOR Pathway Based on Network Pharmacology and Experimental Verification”

**DOI:** 10.1155/mi/9832083

**Published:** 2026-01-09

L. Tang, C. Zhao, J. Zhang, T. Dong, H. Chen, T. Wei, J. Wang, and W. Yang, “Discussion on the Mechanism of Gandoufumu Decoction Attenuates Liver Damage of Wilson’s Disease by Inhibiting Autophagy through the PI3K/Akt/mTOR Pathway Based on Network Pharmacology and Experimental Verification,” *Mediators of Inflammation*, 2023, https://doi.org/10.1155/2023/3236911.

In the article, there are errors in Figure 3c. Specifically:

• The Gandoufumu High‐dose and Middle‐dose panels in the last row of the Sirius Red staining images are duplicated.
• The Normal and Penicillamine panels in the last row of Sirius Red staining were 100x, whereas the figure legend specifies 400x.

The correct Figure 3 is shown below:

Figure 3Effect of GDFMD on serum liver function, liver oxidative stress, pathological liver changes, and serum copper content of mouse models of WD. Serum levels of ALT, AST, TP, and ALB (a); liver oxidative stress of SOD, GSH‐Px, MDA, and ROS was measured by ELISA assays (b). Representative photomicrographs of morphological changes (c) in HE (400x), Masson (400x), and Sirius red (400x) staining in liver tissues, scoring of liver histology (d), and quantification of Sirius red staining area (e) of mice from the control, model, Gandoufumu (high dose, low dose, and middle dose), and penicillamine groups. Serum copper content was determined by copper assay kit (f). Data are depicted in terms of mean ± SD (*n* = 10).  ^∗^ *p* < 0.05 compared with the normal group; ^*△*^ *p* < 0.05 compared with the model group.

**Figure:**
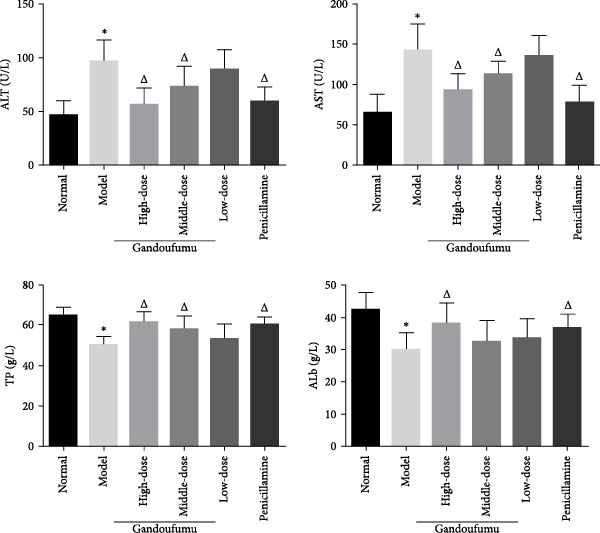
(a)

**Figure:**
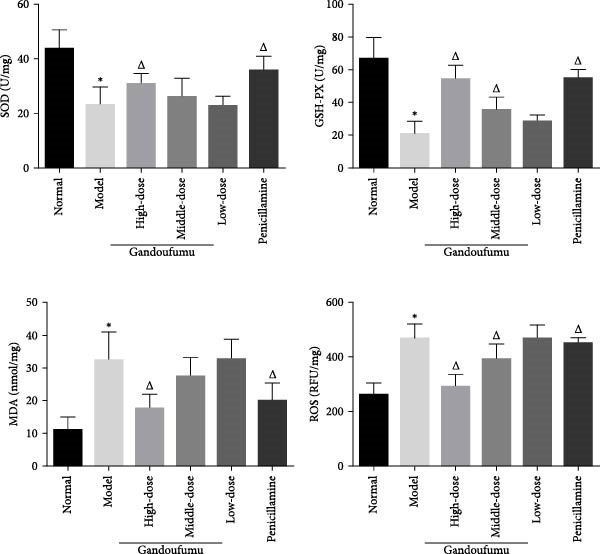
(b)

**Figure:**
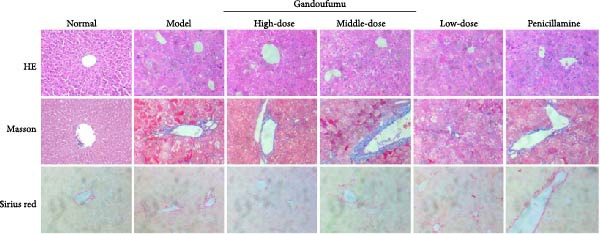
(c)

**Figure:**
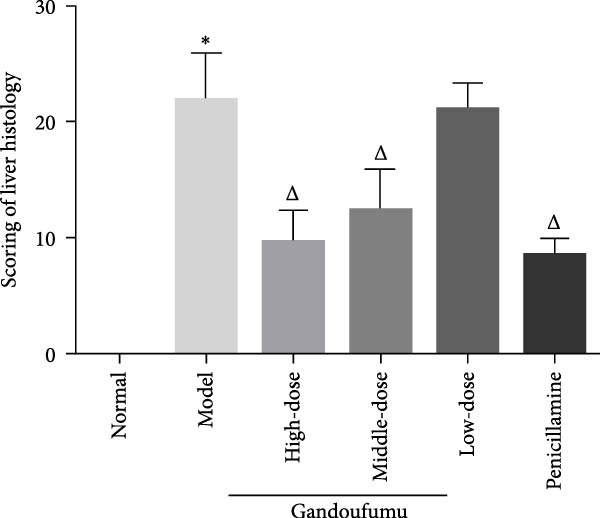
(d)

**Figure:**
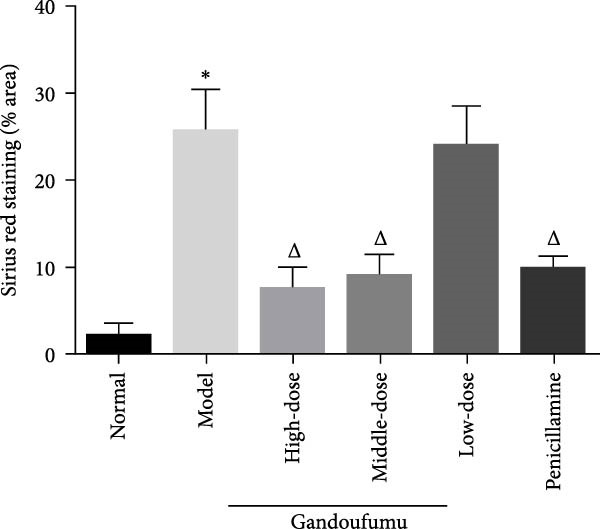
(e)

**Figure:**
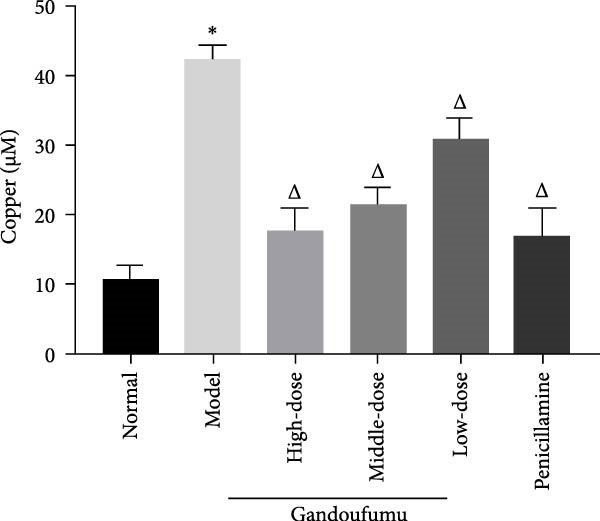
(f)

We apologize for these errors.

